# Does intra-articular injection of adipose-derived stem cells improve cartilage mass? A case report using three-dimensional image analysis software in knee osteoarthritis

**DOI:** 10.1186/s13256-021-03186-6

**Published:** 2021-12-18

**Authors:** Ayano Kuwasawa, Kotaro Nihei

**Affiliations:** Saitama Cooperative Hospital, 1317 Kizoro, Kawaguchi, Saitama 333-0831 Japan

**Keywords:** Case report, Knee osteoarthritis, Adipose-derived stem cell, Mesenchymal stem cells, Magnetic resonance imaging, Act on issue repair, Quantification of cartilage

## Abstract

**Background:**

Mesenchymal stem cells are currently a research focus because of the possibility of cartilage regeneration through several mechanisms, including mesenchymal stem cell sheets. However, there are no published reports visualizing cartilage in three dimensions. Here, we report a case of improved cartilage volume. We purified and cultured adipose-derived mesenchymal stem cells and then performed adipose-derived mesenchymal stem cell therapy by directly injecting these cells into the articular cartilage. Cartilage was quantitatively evaluated before and after injection using three-dimensional image analysis software based on the magnetic resonance imaging.

**Case presentation:**

The patient, a 55-year-old Japanese woman, experienced pain in both knees and was diagnosed with osteoarthritis of the knee. We performed adipose-derived mesenchymal stem cell therapy in both knees at our hospital and quantitatively evaluated cartilage before and after the treatment using the three-dimensional image analysis software “SYNAPSE VINCENT”.

**Conclusions:**

Preoperatively, the cartilage defect area was 33.59 mm^2^ in the femur and 122.31 mm^2^ in the tibia; however, 12 months postoperatively, it improved to 13.59 mm^2^ and 51.43 mm^2^, respectively. Furthermore, the preoperative femur and tibia volumes were 9.58 mL and 3.82 mL, respectively; however, 12 months postoperatively, these values improved to 10.00 mL and 4.17 mL, respectively. For the quantitative analysis of cartilage, SYNAPSE VINCENT visualizes the state of cartilage in a high-definition three-dimensional image, which is excellent for understanding the state of the disease and explaining it to the patient. Although SYNAPSE VINCENT can only analyze the thickness of cartilage, and the reproducibility of the error is debatable, SYNAPSE VINCENT would be useful as a clinical tool for regenerative medicine. We have shown in this case report the promising effects of adipose-derived stem cell intraarticular injections in treating osteoarthritis and the use of new diagnostic instruments.

## Background

Osteoarthritis (OA), which is different from trauma, is a disease caused by dysplasia and malalignment. In OA, deterioration and wear of cartilage, which are associated with synovitis, progress over time. The number of patients with OA of the knee in Japan (diagnosed by X-ray) is estimated to be about 25.30 million (8.6 million males and 16.7 million females), and the number of symptomatic patients is estimated to be about 8 million (a large-scale epidemiologic survey, ROAD) [1]. In addition, 11% of the main causative diseases requiring care for those aged 65 years or older were caused by joint diseases [2]. OA reduces the patients’ quality of life and daily living activities, leading to the shortening of healthy life expectancy.

According to the guidelines, the optimal management of OA of the knee requires a combination of nondrug and drug therapies [3]. However, regenerative medicine cell therapy, including platelet-rich plasma (PRP) therapy, has recently become widespread and gathered attention as the third type of treatment. As a method of using somatic stem cells, we perform adipose-derived mesenchymal stem cell (ASC) therapy in the hospital, where adipose-derived mesenchymal stem cells (MSCs) are purified, cultured, and injected into the joint. To initiate regenerative medicine using ASCs, a medical institution is required to submit “Application for Regenerative Medicine based on the Article 4, paragraph 1, of the Act on the Safety of Regenerative Medicine” to the Japanese Ministry of Health, Labour and Welfare in Japan. At our hospital, our application protocol, titled “Joint treatment by administration of adipose tissue-derived stem cells”, has received approval since April 2018.

Transplantation of ASCs is one of mesenchymal stromal stem cell-based therapies. This can be performed in two ways: one is a method of extracting and culturing only ASCs for transplantation, called adipose-derived MSC; another is a method of treating adipose tissue with an enzyme to separate a stromal vascular cell group from other fat cells, which is then used for transplantation, called stromal vascular fraction (SVF). Although SVF is not common in Japanese hospitals, a cohort study of patients with knee OA who underwent ASC or SVF showed that there were no serious side effects in either group and that ASC groups had less adverse events associated with fat collection [4]. This is likely due to the fact that ASCs can be cultured by collecting a small amount of fat with local anesthesia. Additionally, compared with SVF, though it takes longer to prepare (about 6 weeks), ASCs can be cryopreserved. Indeed, the survival rate of ASCs from freeze to thaw is 96.7% when frozen and 83.5% when thawed; thus, more than 80% of stem cells can act in the joint. Moreover, by injecting MSCs, a factor secreted by stem cells affects the environment in the joint and is expected to act on tissue repair (paracrine effect).

About 20 mL of adipose tissue used for ASC preparation is collected from the patient by a clean operation using a cannula and syringe at Saitama cooperative hospital. The syringe filled with the collected adipose tissue is enclosed in a biohazard bag, packed with a cushioning material, and then shipped to the CellSource Regenerative Medicine Center, a manufacturer of specific cell processed products in Shibuya-ku, Tokyo. After the subculture, ASCs are delivered to our hospital in a frozen state on the day of their scheduled administration and used for the treatment of OA once thawed.

Quantification of cartilage was performed before and after treatment using Fujifilm’s 3D image analysis system “SYNAPSE VINCENT,” which can convert 2D images produced by a magnetic resonance imaging (MRI) scan into high-quality 3D images, and used to confirm the adaptation of autologous cartilage during the transplantation of ASCs.

This is the first case report of ASC therapy for a patient with knee OA at Saitama cooperative hospital.

## Case presentation

Patient: a 55-year-old Japanese female.

None of the factors contributing to lateral compartment knee disease, such as obesity, complications, or psychiatric disorders, were present in the patient. During hyaluronic acid (HA) treatment, although we prescribed strength training to the patient, she did not perform it frequently enough because of severe pain. As the knee pain was caused by tripping while playing tennis, the possibility of trauma cannot be ruled out.

### Medical history

In 2013, due to pain in both knees, she visited our hospital and was diagnosed with OA of the knee.

In December 2017, while playing tennis, the right knee developed a knee collapse. She was examined at the hospital, and an MRI of the right knee was performed. Horizontal dissection of the lateral meniscus and cartilage defect on the lateral condyle of the femur were observed.

Since 2018, injections of hyaluronic acid have been administered every 2 weeks, but joint edema and pain recurrence have been remitted.

The patient had already undergone HA treatment and received multiple steroid joint injections; however, these interventions were not very effective. Moreover, the patient was unwilling to undergo these treatments. The patient was aware that our hospital would start providing regenerative medicine treatments and had been waiting for a year before the start of the treatment. During that period, the patient received HA treatment, which proved ineffective. Therefore, the patient requested ASC treatment in April 2018.

**Fig. 1:**
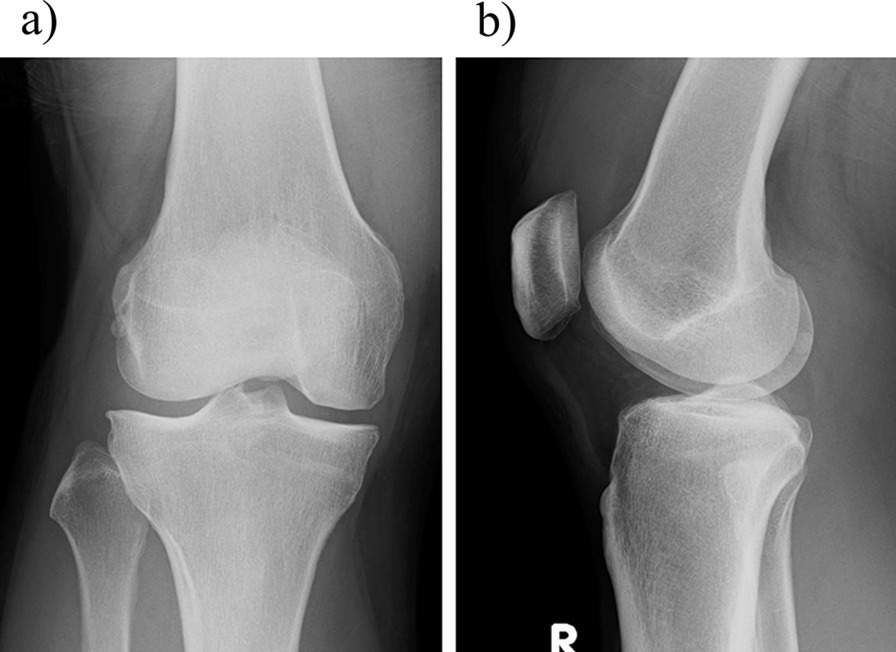
The AP and lateral radiograph of the right knee in April 2018. **a** AP radiograph, **b** lateral radiograph

In April 2018, MRI of the right knee was performed. A cartilage defect was found in the external condyle of the femur, and the patient desired ASC transplantation. The range of motion of the right knee is 0–145. Anteroposterior (AP) and lateral radiograph of the right knee are shown in Fig. 1.

On 28 April 2018, 20 mL ASCs were collected from the abdomen.

On 11 June 2018, the first ASC transplantation was performed on the right knee.

On 2 July 2018, although the effect was experienced after 1 week of treatment, the pain recurred when the subject moved violently.

On 6 August 2018, the patient was in a good condition. When the Timed Up and Go Test was conducted, it showed improvement from 10 seconds to 7 seconds.

On 10 September 2018, the patient was in a good condition. She resumed playing tennis and was living almost without pain.

On 10 December 2018, an MRI was performed. There was no pain even when the subject jumped on one leg and a repaired cartilage defect was observed.

On 22 April 2019, the second ASC transplant was performed on the right knee and the first ASC transplant was performed on the left knee.

On 22 June 2019, there was a slight fever on the day of the procedure.

On 5 August 2019, the patient was in a good condition. It became possible to assume a sitting position.

## Results

**Fig. 2:**
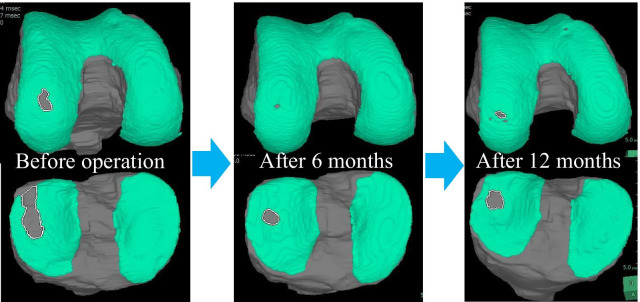
Clinical course of cartilage area evaluated by MRI (3D image rendered by SYNAPSE VINCENT)

**Fig. 3:**
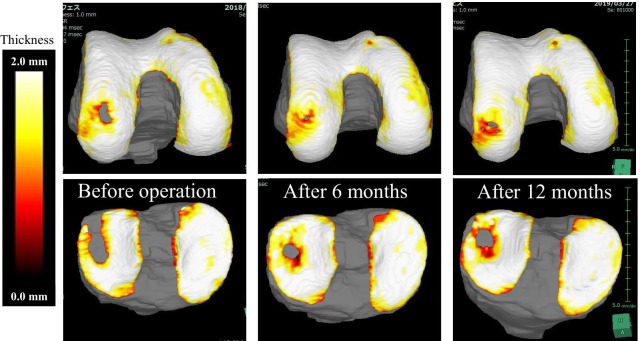
Clinical course of cartilage thickness evaluated by MRI (3D image rendered by VINCENT)

Before the operation, the cartilage defect area was 33.59 mm^2^ in the femur and 122.31 mm^2^ in the tibia; however, 6 months after the operation, it improved to 4.59 mm^2^ in the femur, 34.48 mm^2^ in the tibia, and 12 months after the operation, it improved to 13.59 mm^2^ in the femur and 51.43 mm^2^ in the tibia. The bone volume before the procedure was 9.58 mL for the femur and 3.82 mL for the tibia; however, 6 months after the procedure, it improved to 10.36 mL for the femur and 4.00 mL for the tibia, and 12 months after the procedure, it improved to 10.00 mL for the femur and 4.17 mL for the tibia (Figs. 2, 3, Table 1).

**Table 1:** Change of cartilage defect area and cartilage volume

	Before operation	After 6 months	After 12 months
Cartilage defect area (mm^2^)			
Femur	33.59	4.59	13.59
Tibia	122.31	34.48	51.43
Cartilage volume (mL)			
Femur	9.58	10.36	10.00
Tibia	3.82	4.00	4.17

## Discussion and conclusions

In this case, ASC therapy not only reduced pain but also regenerated the cartilage defect. The side effects were temporary and minor. The reason for the temporary and minor side effects was considered to be the minimal invasion of fat collection and the low burden on the patient.

The reason for regeneration of the cartilage defect area in this case is considered to be that the paracrine effect of the stem cells affected existing surrounding cells in the joint and repaired cartilage around the defect area. In addition, conditions that enable repair of a cartilage defect include the presence of chondrocytes (a limited defect), a small defect, good alignment, and low mechanical stress.

Although there is a published report stating that a capsule was formed by PRP and stem cell transplantation, there is no published report that states that cartilage regeneration was observed by stem cell transplantation in humans. This case shows an increase in the amount of cartilage by directly injecting MSCs into joints.

It is believed that the results of this study were obtained due to the usefulness of SYNAPSE VINCENT as a diagnostic method, our technical skills, and the culture methods of ASCs. SYNAPSE VINCENT enables the quantitative analysis of cartilage, providing 3D visualization of the state of cartilage in an easy to understand manner, which is excellent for determining the state of the disease and explaining it to the patient. SYNAPSE VINCENT has some challenges. Only the thickness of cartilage can be analyzed from the images, and qualitative changes cannot be assessed. Therefore, it cannot distinguish vitreous cartilage from fibrocartilage from the images. Also, the reproducibility of errors is debatable. Despite the above issues, SYNAPSE VINCENT will be useful as a clinical tool for regenerative medicine.

We have shown in this case report the promising effects of adipose-derived stem cell intraarticular injections in treating osteoarthritis and the use of new diagnostic instruments. However, the long-term effects need to be further investigated.

## Data Availability

Not applicable.

## Abbreviations

3D: Three-dimensional
ASC: Adipose-derived mesenchymal stem cells
AP: Anteroposterior
MRI: Magnetic resonance imaging
MSC: Mesenchymal stem cells
OA: Osteoarthritis
PRP: Platelet-rich plasma
SVF: Stromal vascular fraction
